# YouTube as a source of information for stroke rehabilitation: a cross-sectional analysis of quality and reliability of videos

**DOI:** 10.1007/s00296-025-05832-4

**Published:** 2025-03-22

**Authors:** Meirgul I. Assylbek, Olena Zimba, Ahmet Akyol, Marlen Yessirkepov, Burhan Fatih Kocyigit

**Affiliations:** 1 Department of Neurology, Psychiatry, Neurosurgery and Rehabilitation, South Kazakhstan Medical Academy, Shymkent, Kazakhstan; 2 Department of Social Health Insurance and Public Health, South Kazakhstan Medical Academy, Shymkent, Kazakhstan; 3 Medical Center ‘’Mediker’’, Shymkent, Kazakhstan; 4 Department of Clinical Rheumatology and Immunology, University Hospital in Krakow, Krakow, Poland; 5 National Institute of Geriatrics, Rheumatology and Rehabilitation, Warsaw, Poland; 6 Department of Internal Medicine N2, Danylo Halytsky Lviv National Medical University, Lviv, Ukraine; 7 Department of Physical Medicine and Rehabilitation, Faculty of Medicine, Gaziantep Islam Science and Technology University, Gaziantep, Türkiye; 8 Department of Biology and Biochemistry, South Kazakhstan Medical Academy, Shymkent, Kazakhstan; 9 Department of Physical Medicine and Rehabilitation, University of Health Sciences, Adana Health Practice and Research Center, Adana, Türkiye

**Keywords:** Stroke rehabilitation, Neurological rehabilitation, Social media, Internet, Information science

## Abstract

**Introduction:**

Due to YouTube’s meteoric rise in popularity, the quality and reliability of health-related videos on YouTube are being questioned, particularly in specialized fields like stroke rehabilitation. This research aimed to assess the quality and reliability of YouTube videos relevant to stroke rehabilitation.

**Method:**

Video listing was conducted on December 17, 2024, using the keywords “Stroke Rehabilitation”, “Stroke Physical Therapy”, “Stroke Neurophysiotherapy”, and “Stroke Physical Therapy Techniques” as query terms. A final sample of 72 videos was selected upon completion and evaluated according to inclusion and exclusion criteria. The Global Quality Scale (GQS), Modified DISCERN Questionnaire, JAMA Benchmark Criteria, and Patient Education Materials Assessment Tool for Audio/Visual Materials (PEMAT-A/V) were among the evaluation tools used to analyze each video. Researchers captured the videos’ fundamental components and compared the quality classifications.

**Results:**

Of the 72 videos examined, 29.2% (*n* = 21) were categorized as low quality, 20.8% (*n* = 15) as intermediate level, and 50% (*n* = 36) as high quality. Videos generated by academic medical centers (77.8%) and nonphysician healthcare professionals (59.4%) were primarily of high quality, while videos from independent users (100%) and TV channels (66.7%) displayed the lowest quality. Significant differences were observed when comparing quality groups based on daily views, likes, and comments (*p* < 0.05). The lowest scores were detected in the low-quality group. Significant correlations were identified between GQS and other evaluative instruments (*p* < 0.001), indicating consistency across evaluation frameworks.

**Conclusion:**

YouTube possesses considerable potential as an instructional tool for stroke rehabilitation. The inconsistency in video quality underscores the necessity for enhanced content control, editing, and the advocacy of high-quality, evidence-based resources. Promoting collaboration among academics, healthcare professionals, and content producers could augment the platform’s instructional efficacy.

**Supplementary Information:**

The online version contains supplementary material available at 10.1007/s00296-025-05832-4.

## Introduction

The internet has revolutionized access to health-related information, serving as a crucial resource for patients, carers, and medical professionals [1]. As digital devices become more prevalent and internet access expands, individuals turn to online platforms for information regarding medical issues, treatment alternatives, and rehabilitation approaches [2]. This trend represents a change towards patient empowerment, in which people actively participate in understanding their health and discovering ways to improve their well-being. The internet’s ease of use and variety of materials make it the favored platform for individuals searching for health-related information [3, 4].

YouTube has become a favored source of health-related information among the numerous available platforms. YouTube provides a fascinating and accessible platform for disseminating information, hosting millions of videos covering various subjects. The platform’s visual and audio aspects allow consumers to comprehend complicated healthcare subjects that would otherwise be challenging to understand solely through text [5, 6]. YouTube has a variety of health-related videos, including expert seminars, patient experiences, and educational guides, offering a distinctive chance to enhance health literacy. However, the uncontrolled nature of YouTube and the lack of editing support prompt concerns over the quality and accuracy of the information presented [7].

Stroke rehabilitation is an essential stage in the recovery process for individuals who suffered a stroke. This procedure employs a multidisciplinary strategy to restore the physical, mental, and emotional capacities affected by the incident [8]. In stroke rehabilitation, supporting sources, including instructional videos and audio recordings, are essential for improving adherence to physical therapy and facilitating comprehension [9]. Visual material can potentially instruct carers on safe practices, guide patients through appropriate movement approaches, and offer motivational tools to sustain participation in the rehabilitation course [10]. Therefore, stroke survivors can benefit substantially from YouTube and similar platforms, which provide readily available and user-friendly information to supplement conventional rehabilitation methods [11].

Emerging evidence points to a complicated connection between rheumatic disorders and cerebrovascular health. Chronic systemic inflammation, endothelial dysfunction, and immunity-related vascular damage in rheumatic diseases may raise the risk of stroke and impact post-stroke healing pathways. Given these concerns, rehabilitation procedures for stroke patients with underlying rheumatic disorders necessitate a more individualized approach that takes into account disease-related restrictions, fatigue, and potential drug interactions [12, 13].

This research evaluates stroke rehabilitation videos on YouTube. The first aim is to identify sources to reach high-quality videos by analyzing their features. Additionally, we aim to draw illuminating findings by comparing video parameters across quality categories. Lastly, it seeks to shed light on how effectively YouTube disseminates information about stroke rehabilitation.

## Methods

The video inspection was held on December 17, 2024, with the keywords “Stroke Rehabilitation”, “Stroke Physical Therapy”, “Stroke Neurophysiotherapy” and “Stroke Physical Therapy Techniques” as query terms. The MeSH terms were used to determine the search phrases. Because YouTube places a premium on providing users with tailored results, all cookies and history were erased. The goal was to lessen the impact of past internet use. Before conducting the search, the Google Chrome browser was set to incognito mode to ensure anonymity for users. The listing was created using the default setting of “relevance-based ranking,” which mirrors the routine actions of the typical platform user [14, 15]. A typical internet user accesses only a restricted segment of the listing. This has been substantiated by previous research. Therefore, the initial 50 videos for each search term were used in the assessment [16, 17]. The exclusion parameters were defined as follows: (1) videos in languages other than English, (2) repeated videos, (3) irrelevant videos, (4) videos shorter than 1 min or more than 60 min, and (5) videos having auditory or image defects. Videos under 1 min were excluded because they lacked complexity and thoroughness for providing valuable educational material, especially with the elaborate healthcare process of stroke rehabilitation. These concise videos may prioritize swift summaries, marketing materials, or incomplete interpretations that do not achieve the goal of thorough information dissemination. In contrast, videos over 60 min were removed to align with YouTube’s typical user engagement patterns and ensure practical application. Online viewers, particularly those searching for medical information, tend to disengage from excessively long videos, finding them overwhelming, monotonous, or challenging to follow. In stroke rehabilitation, where patients and carers need clear, brief, and practical content, lengthy videos may cause cognitive overload and decreased retention of essential details. By establishing this criteria, we targeted videos more likely to be thoroughly viewed, retained, and realistically implemented by the intended audience.

The first step of the video assessment process was for two researchers to score the videos separately. Discrepancies were detected by comparing the independent ratings. Regarding the videos that had distinctions, a third researcher ultimately concluded. The agreement between scores was documented using Cohen’s kappa coefficient [18].

### Video parameters

The subsequent fundamental parameters were acquired via YouTube:

- Count of views, likes, and comments.
- Duration of the video (seconds).
- Interval between video upload date and listing date (days).
- Daily metrics of views, likes, and comments.

Based on the method of formatting, the videos were divided into four groups: (1) those with sole narrators, (2) those with a focus on the experiences of patients, (3) those with animations, and (4) those with slides to present.

### Sources

Video sources were classified into the following categories:

- Physician.
- Nonphysician health care professional.
- Academic medical centers.
- Nonacademic healthcare facilities.
- TV channels.
- Nonprofit charities or foundations.
- Independent user.

### Content evaluation

The Global Quality Scale (GQS), an established method for evaluating the usefulness and efficacy of educational resources found online, was implemented to measure the quality. Parts one through five make up the GQS. The potential scores are from 1 (very low) to 5 (very high). Inconsistency and significant gaps in the data are indicated by a score of 1. A score of 5 indicates a high level of consistency, which is highly beneficial. From 4 to 5 for high-quality videos to 3 for intermediate quality, videos rated 1 or 2 are considered low Quality [19, 20].

Researchers used the modified DISCERN instrument to measure reliability. Clarity, intelligibility, bias, objectivity, and the inclusion of references and supplemental resources are among the multiple criteria that this tool checks. Using dichotomous queries, this approach provides one point to positive responses and zero to failed responses. The most outstanding score that can be achieved is 5 [21].

The JAMA Benchmark Criteria serve as a framework for evaluating the accuracy and quality of online health data. These criteria assess fundamental elements that ensure the reliability of internet-based items, including ownership, authentication, disclosure, and currency [16].

Visual and auditory medical educational sources can be systematically evaluated regarding their understandability and actionability by employing the Patient Education Materials Assessment Tool for Audio/Visual Materials (PEMAT-A/V). By focussing on factors like organizational structure, terminology, clarity, and imagery, understandability evaluates how easily individuals may absorb knowledge. Actionability assesses whether the sources of information clearly outline tasks that patients can undertake to address the issue at hand. The percentage serves to denote the scores [22].

### Statistical analysis

The Statistical Package for the Social Sciences (SPSS) software, version 29.0 (SPSS Inc., Chicago, IL, USA), was implemented for statistical analysis. The normality of the data distribution was checked using the Shapiro-Wilk test before the analyses. Medians, frequencies (n), and percentages (%) were utilized to express the results. The dataset was sorted into three quality categories, and the Kruskal-Wallis test was used to compare them. Researchers used Spearman’s rho test for correlation analyses. Also, to find out the degree of agreement, researchers relied on the Kappa coefficient. For statistical significance, a p-value below 0.05 was considered adequate.

## Results

**Fig. 1:**
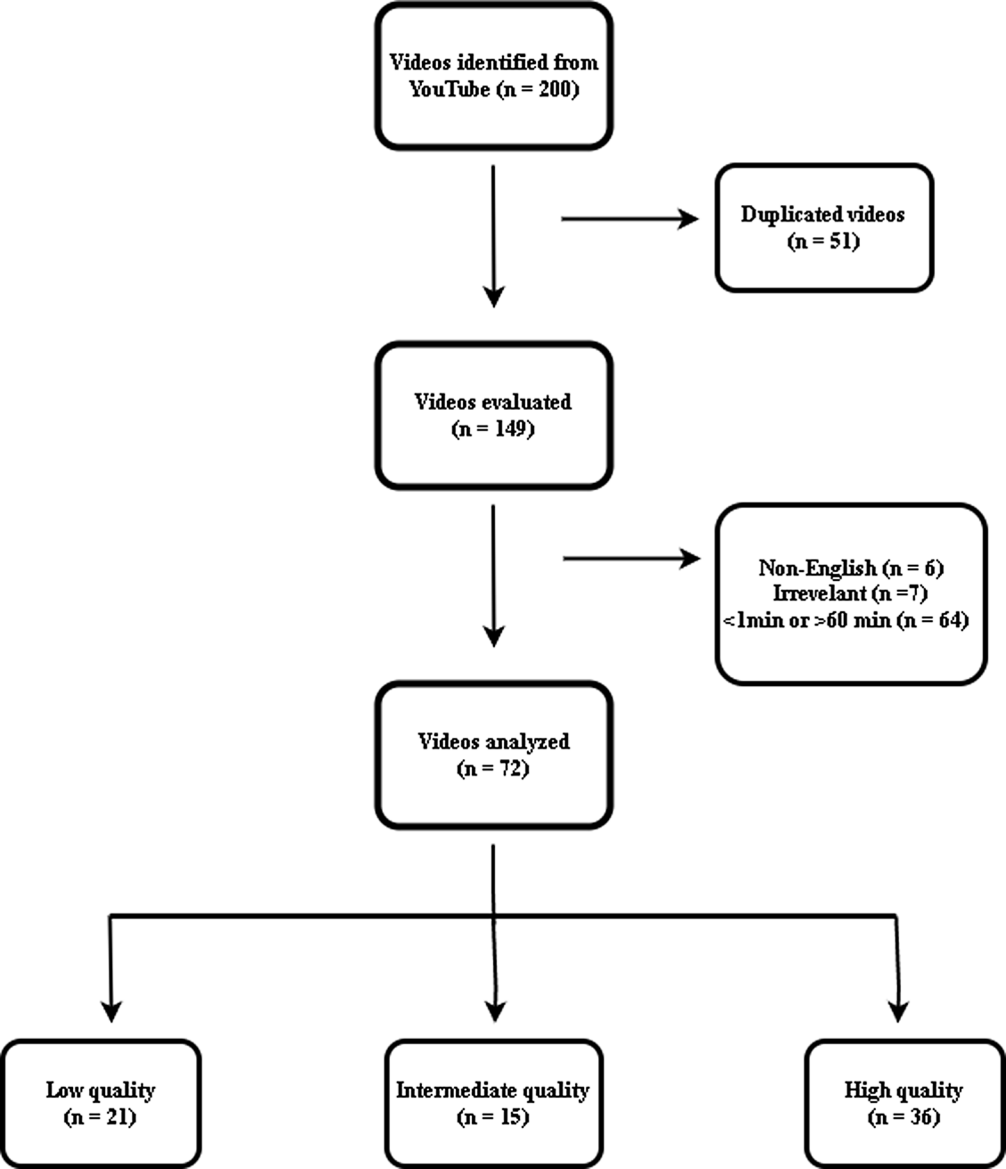
Visualization of the inclusion and exclusion process of videos

A list of the most relevant 50 videos for each search phrase was compiled. For this analysis, 200 published videos were assessed; nevertheless, 128 were disqualified according to the criteria, allowing 72 videos to be considered. More information regarding the sample procedure is presented in Fig. 1. The median duration of the videos was 438.50 (61–3081) seconds. The median number of views, likes, and comments were 97,242 (51–1905604), 1200 (0–20000), and 64.50 (0–414), respectively. Among the videos, 80.6% (*n* = 58) were presented with narrators only, 6.9% (*n* = 5) with patient experiences, 4.2% (*n* = 3) with animations and 8.3% (*n* = 6) with slide presentations. The primary features of the videos are outlined in Table 1.

**Table 1:** Primary features of the videos

Video features	
Duration (seconds)^*^	438.50 (61–3081)
Number of views^*^	97,242 (51–1905604)
Number of likes^*^	1200 (0–20000)
Number of comments^*^	64.50 (0 - 414)
Days since upload^*^	1709 (27 - 4978)
Views per day^*^	66.4 (0.1– 477.8)
Likes per day^*^	0.9 (0– 7.2)
Comments per day^*^	0.1 (0– 0.5)
**Presentation method (n; %)**	
Video containing only narrator(s)	58 (80.6)
Video containing patient experiences	5 (6.9)
Animation	3 (4.2)
Narrating with a slide presentation	6 (8.3)

^*^ Data are expressed as median (minimum-maximum)

Based on the GQS rankings, the videos were split into three categories: low, intermediate, and high quality. Out of the total number of videos, 29.2% (*n* = 21) were deemed low quality, 20.8% (*n* = 15) were deemed intermediate, and 50% (*n* = 36) were deemed high quality. Video sources were categorized based on their quality. Academic medical centers and nonphysician health care professionals make up the bulk of the sources that supply high-quality videos (77.8% and 59.4%, respectively). However, according to Table 2, the sources that provided the lowest quality videos were independent users (100%) and TV channels (66.7%).

**Table 2:** Categorization of the videos according to sources, N (%)

Source	Low quality	Intermediate quality	High quality	Total
Physician	0 (0)	0 (0)	0 (0)	0
Nonphysician health care professional	8 (25)	5 (15.6)	19 (59.4)	32
Academic medical centers	0 (0)	2 (22.2)	7 (77.8)	9
Nonacademic health care facilities	6 (37.5)	4 (25)	6 (37.5)	16
TV channels	2 (66.7)	1 (33.3)	0	3
Nonprofit charities or foundations	2 (22.2)	3 (33.3)	4 (44.5)	9
Independent user	3 (100)	0 (0)	0 (0)	3

n: number, %: percentage

Significant differences were observed when comparing quality groups based on daily views, likes, and comments (*p* < 0.05). The lowest scores were detected in the low-quality group (Table 3).

**Table 3:** Comparison of the video parameters between the low-quality, intermediate, and high-quality groups

	Low quality	Intermediate quality	High quality	*p*
Views per day	7.69 (0.07–328.76)	67.82 (9.57–423.55)	114.44 (1.55–477.81)	< 0.001
Likes per day	0.05 (0–7.18)	0.83 (0.05–6.12)	2.30 (0–6.69)	0.001
Comments per day	0 (0–0.31)	0.08 (0–0.41)	0.05 (0–0.52)	0.004

Correlation analyses were conducted between GQS and other video assessment tools, revealing statistically significant correlations (*p* < 0.001 for all and rho = 0.859 for Modified DISCERN Questionnaire, rho = 0.833 for JAMA Benchmark Criteria, rho = 0.816 for PEMAT-A/V Understandability and rho = 0.844 for PEMAT-A/V Actionability). In addition, researchers analyzed the correlations between the video parameters and the ratings on the video evaluation tools. Results showed positive correlations between the video duration and ratings on the GQS, Modified DISCERN Questionnaire, JAMA Benchmark Criteria, PEMAT-A/V Understandability, and PEMAT-A/V Actionability (*p* < 0.001). Views per day and likes per day data were significantly and positively correlated with video evaluation tools (*p* < 0.05) (Table 4).

**Table 4:** Correlation analysis between content scores and video parameters

	GQS	Modified DISCERN Questionnaire	JAMA Benchmark Criteria	PEMAT-A/V Understandability	PEMAT-A/V Actionability
Video duration	**0.662** ^**a**^	**0.504** ^**a**^	**0.505** ^**a**^	**0.485** ^**a**^	**0.595** ^**a**^
Days since upload	0.085	-0.015	0.041	-0.004	0.010
Views per day	**0.319** ^**b**^	**0.245** ^**b**^	**0.258** ^**b**^	**0.249** ^**b**^	**0.248** ^**b**^
Likes per day	**0.339** ^**b**^	**0.262** ^**b**^	**0.254** ^**b**^	**0.302** ^**b**^	**0.293** ^**b**^
Comments per day	**0.281** ^**b**^	0.178	0.141	0.213	0.180

GQS: Global Quality Scale; JAMA: Journal of the American Medical Association; PEMAT-A/V: Patient Education Materials Assessment Tool for Audio/Visual Materials

^a^ indicates *p* < 0.01; ^b^ indicates *p* < 0.05

A Kappa coefficient of 0.81 was determined.

## Discussion

**Fig. 2:**
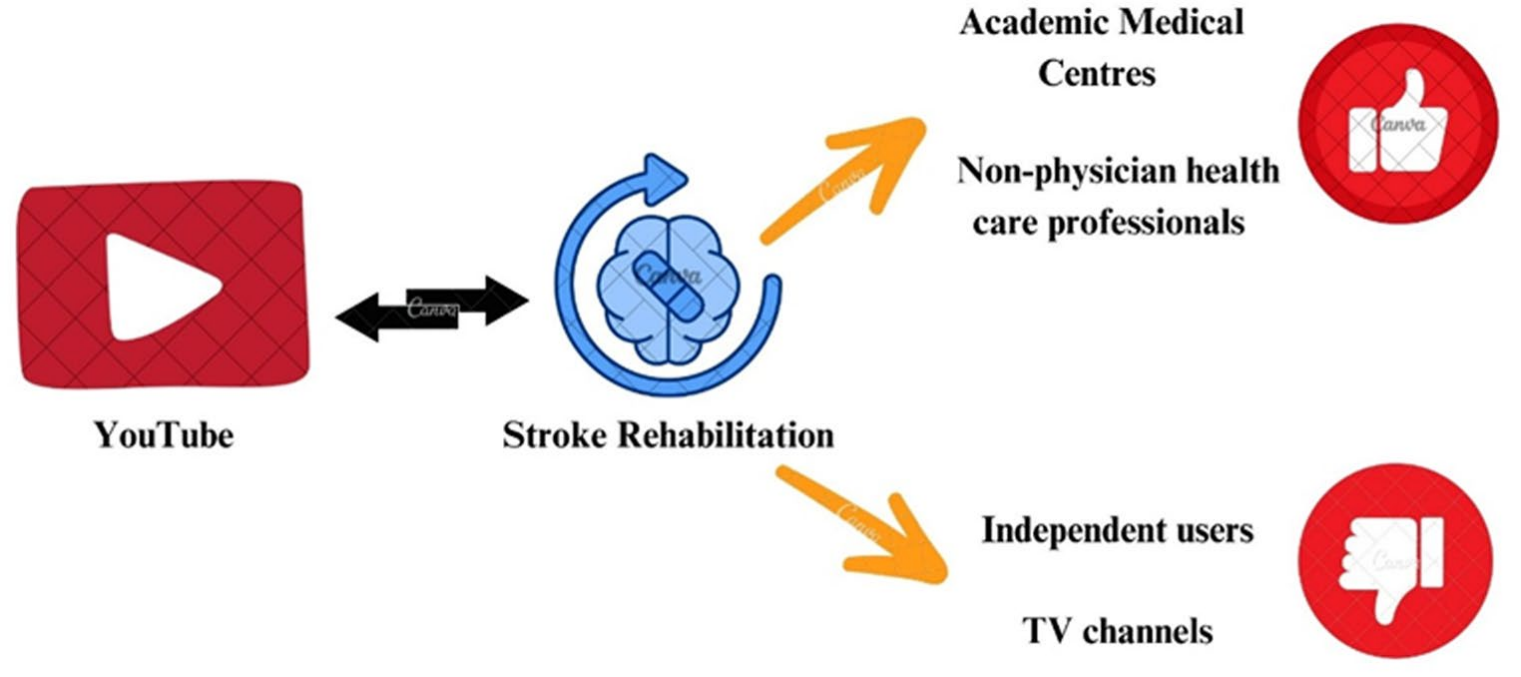
High and low-quality video sources on YouTube videos related to stroke rehabilitation

The investigation of stroke rehabilitation videos on YouTube yields substantial knowledge of the quality and features of this popular platform. Primary findings reveal that although YouTube offers diverse videos on stroke rehabilitation, considerable variation exists in their quality. Academic medical centers and nonphysician healthcare professionals were the predominant suppliers of high-quality videos, while independent users and TV channels were linked to poor content (Fig. 2).

The widespread use of narrators in stroke rehabilitation videos highlights the necessity of diversity in information delivery forms. While narrators offer comprehensive clarifications, animations, and patient stories have distinct advantages, such as improving visual learning and establishing emotional engagement. Expanding the usage of different formats can increase participation and accessibility for a broad audience [23].

The findings indicate substantial variations in the quality of stroke rehabilitation materials on YouTube. High-quality videos comprised half of the examined content, showing an extensive presence of valuable materials. However, approximately one-third of the videos were of low quality, demonstrating a diversity in informative standards. Notably, academic medical centers and nonphysician healthcare professionals are identified as the leading sources of high-quality videos, demonstrating their dedication to evidence-based and reliable instruction. On the other hand, independent users and TV channels were linked to the poorest quality videos. This sharp difference emphasizes the need for trusted sources to provide dependable information, as well as the hazards posed by uncontrolled or inexperienced contributors. These findings underline the necessity of directing users to trustworthy sources and the need for stronger content management, editing mechanisms, and quality assurance systems on the platform [5, 24]. To enhance the effectiveness of high-quality content, it is essential to aggregate all trustworthy YouTube videos on stroke rehabilitation onto specialized sites overseen by local and international stroke societies. Centralized databases can be reliable resources for patients and healthcare professionals, providing consistent access to trustworthy educational content [25].

Significant differences across quality groups were based on daily views, likes, and comments. Low-quality videos had the poorest values for daily engagement measures involving views, likes, and comments. This suggests that low-quality videos fail to fulfill instructional criteria and struggle to pique users’ interest and maintain engagement. In contrast, higher-quality videos are likely to contain more comprehensive and user-friendly material, increasing their attractiveness and utility. These findings emphasize the possible utility of user engagement measures as indirect markers of video quality [26]. However, depending solely on this data as a measure of high quality may not always be the best strategy.

The significant correlations between the GQS and other video evaluation instruments (Modified DISCERN Questionnaire, JAMA Benchmark Criteria, and PEMAT-A/V) highlight the reliability and validity of these frameworks in assessing health-related material on YouTube. These statistics demonstrate a constant concordance among different tools in detecting videos that adhere to elevated instructional accuracy, reliability, and usefulness requirements. The result underscores the complementing characteristics of varying evaluation approaches, with each instrument targeting distinct facets. Their joint application provides a thorough evaluation.

Video quality and reliability were more directly correlated with duration, with longer videos performing higher on all assessment tools. These significant correlations indicate that longer videos deliver more in-depth explanations, which is critical for stroke rehabilitation instruction. Longer videos are more likely to include elaborate demonstrations, step-by-step guidance, and in-depth explanations, increasing their instructional value [27]. However, lengthy information should be entertaining and well-structured to sustain audience interest and comprehension. These findings support prioritizing video material that balances duration, clarity, and depth, making it more effective for Internet users.

Video assessment instruments were significantly and positively correlated with views per day and likes per day data. This suggests that videos with greater engagement rates are of higher quality, as proven by their performance across various assessment metrics. Consequently, user engagement metrics may function as supplementary indicators of content quality, which would further bolster their value for discovering beneficial sources for stroke rehabilitation.

The article features several limitations, many of which stem from its design and the internal framework of YouTube. First, this article offers a snapshot of YouTube videos. Considering the constantly changing atmosphere of YouTube, the continuous uploading and publishing of new videos, and the fluctuating amount of views, likes, and comments, an identical study undertaken over another time frame may produce different outcomes. Evaluating solely English-language videos ignores videos in other languages, which reach a substantial number of Internet consumers. Using YouTube’s relevance-based ranking algorithm may have biased the selection of videos.

## Conclusion, rheumatic perspectives, and publishing ethics

YouTube is a promising setting for spreading stroke rehabilitation knowledge, with easily accessible and engaging content to supplement established healthcare procedures. However, the substantial variety in video quality highlights the need for more content regulation, editing processes, and the promotion of high-quality sources. Academic medical institutions and nonphysician healthcare professionals were the primary sources of high-quality videos, while independent users and TV channels were associated with poor material. Initiatives to strengthen collaboration between healthcare experts and content developers may improve the production of evidence-based, user-friendly materials. Furthermore, encouraging different video formats—such as animations, patient experiences, story-telling, and guided exercises—may increase the attraction and application of stroke rehabilitation content on YouTube.

Stroke and rheumatic disorders exhibit comparable inflammatory pathways that may heighten stroke risk and affect rehabilitation efficacy. Patients with rheumatic diseases such as rheumatoid arthritis and systemic lupus erythematosus exhibit a heightened risk of stroke attributable to chronic inflammation, endothelial dysfunction, and coagulation irregularities [28, 29]. Appropriate stroke rehabilitation in this demographic requires customized procedures that consider physical constraints, fatigue, and disease activity. Research has emphasized the importance of comprehensive rehabilitation approaches in enhancing recovery outcomes for stroke patients with rheumatic diseases [30]. Due to the possible intricacies of rehabilitation, educational resources on platforms, including YouTube, should offer targeted assistance to meet the requirements of this subgroup, hence enhancing accessibility and efficacy.

Based on the knowledge concerning the positive and negative implications of publishing YouTube posts, it is necessary to maintain video material norms, encourage professionals in critical evaluations, and educate healthcare customers on discerning accurate data from deceptive content. Using artificial intelligence for technical reviews on YouTube may help to improve the foundation of evidence and the general quality of videos. Public health publishers should adhere to established global ethical guidelines and scientific reporting norms to present evidence-based content while safeguarding individual anonymity and security. Like academic papers, incorporating pertinent references, revealing conflicts of interest, and including additional ethical considerations can augment the legitimacy of YouTube content [31].

YouTube can become a more dependable and powerful platform for stroke rehabilitation education by focusing on evidence-based techniques and harnessing cutting-edge technologies. These efforts will not only benefit stroke survivors and their carers but will also pave the way for higher-quality health information on digital platforms.

## Electronic supplementary material

Below is the link to the electronic supplementary material.

Supplementary Material 1

Supplementary Material 2

Supplementary Material 3

Supplementary Material 4

Supplementary Material 5

Supplementary Material 6

Supplementary Material 7

Supplementary Material 8

Supplementary Material 9

Supplementary Material 10

## Declarations

### Disclaimer

Authors state that no part of this manuscript, including the text and graphics, are copied or published elsewhere in whole or in part.

## References

[1] Ducrot P, Montagni I, Nguyen Thanh V, Serry AJ, Richard JB (2021) Evolution of online Health-Related information seeking in France from 2010 to 2017: results from nationally representative surveys. J Med Internet Res 23:e18799. 10.2196/18799 33851927 10.2196/18799 PMC8082381

[2] Le LH, Hoang PA, Pham HC (2023) Sharing health information across online platforms: A systematic review. Health Commun 38:1550–1562. 10.1080/10410236.2021.2019920 34978235 10.1080/10410236.2021.2019920

[3] Kocyigit BF, Akyol A (2021) YouTube as a source of information on COVID-19 vaccination in rheumatic diseases. Rheumatol Int 41:2109–2115. 10.1007/s00296-021-05010-2 34562126 10.1007/s00296-021-05010-2 PMC8475344

[4] Zimba O, Gasparyan AY (2021) Social media platforms: a primer for researchers. Reumatologia 59:68–72. 10.5114/reum.2021.102707 33976459 10.5114/reum.2021.102707 PMC8103414

[5] Koo BS, Kim D, Jun JB (2021) Reliability and quality of Korean YouTube videos for education regarding gout. J Korean Med Sci 36:e303. 10.3346/jkms.2021.36.e303 34811977 10.3346/jkms.2021.36.e303 PMC8608924

[6] Kaplan K, Solak Y (2023) Evaluation of YouTube videos on hepatocellular carcinoma. J Korean Med Sci 38:e50. 10.3346/jkms.2023.38.e50 36808545 10.3346/jkms.2023.38.e50 PMC9941019

[7] Kocyigit BF, Akaltun MS, Sahin AR (2020) YouTube as a source of information on COVID-19 and rheumatic disease link. Clin Rheumatol 39:2049–2054. 10.1007/s10067-020-05176-3 32447603 10.1007/s10067-020-05176-3 PMC7245189

[8] Assylbek MI, Kocyigit BF, Yessirkepov M, Zimba O (2024) Post-stroke rehabilitation in the peri-pandemic COVID-19 era. Rheumatol Int 44:399–411. 10.1007/s00296-023-05520-1 38253904 10.1007/s00296-023-05520-1

[9] Gebreheat G, Goman A, Porter-Armstrong A (2024) The use of home-based digital technology to support post-stroke upper limb rehabilitation: A scoping review. Clin Rehabil 38:60–71. 10.1177/02692155231189257 37469176 10.1177/02692155231189257 PMC10631286

[10] Eames S, McKenna K, Worrall L, Read S (2003) The suitability of written education materials for stroke survivors and their carers. Top Stroke Rehabil 10:70–83. 10.1310/KQ70-P8UD-QKYT-DMG4 14681821 10.1310/KQ70-P8UD-QKYT-DMG4

[11] Denham AM, Baker AL et al (2020) YouTube as a resource for evaluating the unmet needs of caregivers of stroke survivors. Health Inf J 26:1599–1616. 10.1177/1460458219873538 10.1177/1460458219873538 31722610

[12] Huang YC, Lai EC, Liao TC, Weng MY (2024) Evaluating the risk of ischemic stroke at a young age in patients with autoimmune inflammatory rheumatic diseases: a population-based cohort study in Taiwan. Front Immunol 15:1272557. 10.3389/fimmu.2024.1272557 38404587 10.3389/fimmu.2024.1272557 PMC10884215

[13] Sagtaganov Z, Yessirkepov M, Bekaryssova D, Suigenbayev D (2024) Managing rheumatoid arthritis and cardiovascular disease: the role of physical medicine and rehabilitation. Rheumatol Int 44:1749–1756. 10.1007/s00296-024-05651-z 38914772 10.1007/s00296-024-05651-z

[14] Sui W, Sui A, Rhodes RE (2022) What to watch: practical considerations and strategies for using YouTube for research. Digit Health 8:20552076221123707. 10.1177/20552076221123707 36105625 10.1177/20552076221123707 PMC9465614

[15] Zhaksylyk A, Yessirkepov M, Akyol A, Kocyigit BF (2024) YouTube is a source of information on public health ethics. J Korean Med Sci 39:e61. 10.3346/jkms.2024.39.e61 38412608 10.3346/jkms.2024.39.e61 PMC10896704

[16] Etzel CM, Bokshan SL, Forster TA, Owens BD (2022) A quality assessment of YouTube content on shoulder instability. Phys Sportsmed 50:289–294. 10.1080/00913847.2021.1942286 34121601 10.1080/00913847.2021.1942286

[17] Adorisio O, Silveri M, Torino G (2021) Evaluation of educational value of YouTube videos addressing robotic pyeloplasty in children. J Pediatr Urol 17. 10.1016/j.jpurol.2020.12.025.:390.e1-390.e4 10.1016/j.jpurol.2020.12.025 33558173

[18] Luu NN, Yver CM, Douglas JE et al (2021) Assessment of YouTube as an educational tool in teaching key Indicator cases in otolaryngology during the COVID-19 pandemic and beyond: neck dissection. J Surg Educ 78:214–231. 10.1016/j.jsurg.2020.06.019 32646815 10.1016/j.jsurg.2020.06.019 PMC7338020

[19] Ergenç M, Uprak TK (2023) YouTube as a source of information on Helicobacter pylori: content and quality analysis. Helicobacter 28:e12971. 10.1111/hel.12971 36942858 10.1111/hel.12971

[20] Kocyigit BF, Akaltun MS (2019) Does YouTube provide high quality information? Assessment of Secukinumab videos. Rheumatol Int 39:1263–1268. 10.1007/s00296-019-04322-8 31069444 10.1007/s00296-019-04322-8

[21] Korkmaz M, Altin YF, Yagci TF, Korkmaz MD, Akgul T (2024) Is YouTube a reliable and quality source on unilateral biportal endoscopic spine surgery?? A Cross-Sectional study. World Neurosurg 187:e181–e8. 10.1016/j.wneu.2024.04.063 38642831 10.1016/j.wneu.2024.04.063

[22] Karataş L, Utkan Karasu A, Demirsoy N (2024) Is YouTube a sufficient and reliable source to inform patients about cardiac rehabilitation?? A Cross-sectional study. J Cardiopulm Rehabil Prev 44:239–247. 10.1097/HCR.0000000000000864 38875164 10.1097/HCR.0000000000000864

[23] Moulton ST, Türkay S, Kosslyn SM (2017) Does a presentation’s medium affect its message? PowerPoint, Prezi, and oral presentations. PLoS ONE 12:e017877. 10.1371/journal.pone.0178774 10.1371/journal.pone.0178774 PMC5497950 28678855

[24] Kocyigit BF, Nacitarhan V, Koca TT, Berk E (2019) YouTube as a source of patient information for ankylosing spondylitis exercises. Clin Rheumatol 38:1747–1751. 10.1007/s10067-018-04413-0 30645752 10.1007/s10067-018-04413-0

[25] Assadi R, Gasparyan AY (2019) Editing, publishing and aggregating video articles: do we need a scholarly approach?? J Korean Med Sci 30:1211–1212. 10.3346/jkms.2015.30.9.1211 10.3346/jkms.2015.30.9.1211 PMC4553665 26339158

[26] Szmuda T, Talha SM, Singh A, Ali S, Słoniewski P (2021) YouTube as a source of patient information for meningitis: A content-quality and audience engagement analysis. Clin Neurol Neurosurg 202:106483. 10.1016/j.clineuro.2021.106483 33497948 10.1016/j.clineuro.2021.106483

[27] Aydin MA, Akyol H (2020) Quality of information available on YouTube videos pertaining to thyroid Cancer. J Cancer Educ 35:599–605. 10.1007/s13187-019-01502-9 30838529 10.1007/s13187-019-01502-9

[28] Smiyan S, Komorovsky R, Koshak B, Duve K, Shkrobot S (2024) Central nervous system manifestations in rheumatic diseases. Rheumatol Int 44:1803–1812. 10.1007/s00296-024-05679-1 39136787 10.1007/s00296-024-05679-1

[29] Ausserwinkler M, Gensluckner S, Frey V et al (2025) Cerebrovascular risk in rheumatoid arthritis patients: insights from carotid artery atherosclerosis in the paracelsus 10,000 study. Rheumatol Int 45:33. 10.1007/s00296-024-05781-4 39825928 10.1007/s00296-024-05781-4 PMC11742769

[30] Assylbek MI, Zimba O, Yessirkepov M, Kocyigit BF (2024) Healthcare professionals’ knowledge and perceptions of post-stroke rehabilitation in the peripandemic period: an online cross-sectional survey. Rheumatol Int 44:3063–3071. 10.1007/s00296-024-05746-7 39460762 10.1007/s00296-024-05746-7

[31] Zimba O, Gasparyan AY, Qumar AB (2024) Ethics for disseminating Health-Related information on YouTube. J Korean Med Sci 39:e93. 10.3346/jkms.2024.39.e93 38412615 10.3346/jkms.2024.39.e93 PMC10896703

